# Estimate of the incidence of PANDAS and PANS in 3 primary care populations

**DOI:** 10.3389/fped.2023.1170379

**Published:** 2023-09-21

**Authors:** Ellen R. Wald, Jens Eickhoff, Grace E. Flood, Michael V. Heinz, Daniel Liu, Alisha Agrawal, Richard P. Morse, Veronica M. Raney, Aravindhan Veerapandiyan, Juliette C. Madan

**Affiliations:** ^1^ Department of Pediatrics, University of Wisconsin School of Medicine and Public Health, Madison, WI, United States; ^2^ Department of Biostatistics and Medical Informatics, University of Wisconsin School of Medicine and Public Health, Madison, WI, United States; ^3^ Medical Director Clinical Analytics and Reporting, University of Wisconsin, Madison, WI, United States; ^4^ Department of Pediatrics, Children’s Hospital at Dartmouth, Geisel School of Medicine at Dartmouth, Hanover, NH, United States; ^5^ Department of Pediatrics, University of Arkansas for Medical Sciences, Little Rock, AR, United States; ^6^ Department of Neurology, Geisel School of Medicine at Dartmouth, Hanover, NH, United States; ^7^ Department of Psychiatry, University of Arkansas for Medical Sciences, Little Rock, AR, United States; ^8^ Department of Psychiatry, Children’s Hospital at Dartmouth, Geisel School of Medicine at Dartmouth, Hanover, NH, United States; ^9^ Department of Epidemiology, Geisel School of Medicine at Dartmouth, Hanover, NH, United States

**Keywords:** PANS, PANDAS, group A streptococcus, obsessive-compulsive disorder, food restriction, epidemiology

## Abstract

**Objective:**

Pediatric Autoimmune Neuropsychiatric Disorder Associated with Streptococcal infection (PANDAS) and Pediatric Acute-Onset Neuropsychiatric syndrome (PANS) are presumed autoimmune complications of infection or other instigating events. To determine the incidence of these disorders, we performed a retrospective review for the years 2017–2019 at three academic medical centers.

**Methods:**

We identified the population of children receiving well-child care at each institution. Potential cases of PANS and PANDAS were identified by including children age 3–12 years at the time they received one of five new diagnoses: avoidant/restrictive food intake disorder, other specified eating disorder, separation anxiety disorder of childhood, obsessive-compulsive disorder, or other specified disorders involving an immune mechanism, not elsewhere classified. Tic disorders was not used as a diagnostic code to identify cases. Data were abstracted; cases were classified as PANDAS or PANS if standard definitions were met.

**Results:**

The combined study population consisted of 95,498 individuals. The majority were non-Hispanic Caucasian (85%), 48% were female and the mean age was 7.1 (SD 3.1) years. Of 357 potential cases, there were 13 actual cases [mean age was 6.0 (SD 1.8) years, 46% female and 100% non-Hispanic Caucasian]. The estimated annual incidence of PANDAS/PANS was 1/11,765 for children between 3 and 12 years with some variation between different geographic areas.

**Conclusion:**

Our results indicate that PANDAS/PANS is a rare disorder with substantial heterogeneity across geography and time. A prospective investigation of the same question is warranted.

## 1. Introduction

Pediatric autoimmune neuropsychiatric disorder associated with streptococcal infection (PANDAS), believed to be a non-suppurative complication of infection with Group A streptococcus (GAS), was first described in 1998 (1). The existence of PANDAS as a clinical entity distinct from Sydenham chorea, childhood onset obsessive-compulsive disorder (OCD) or tic disorder of childhood has been controversial since its initial description (2, 3). The controversy has focused, at least in part, on the inclusion of tics as a presenting symptom and the relationship of the syndrome to GAS as an instigator event and a cause of exacerbations of neuropsychiatric symptoms. A decade later, the concept of pediatric acute-onset neuropsychiatric syndrome (PANS) was introduced to broaden the scope of this disorder and to allow for provocative events other than GAS (4).

Neither the incidence nor natural history of PANDAS or PANS has been systematically described. In one prospective evaluation performed in a large private pediatric practice in Rochester, NY, children with the acute presentation of neuropsychiatric or behavioral symptoms suggestive of PANDAS were evaluated for evidence of streptococcal infection (5). In this study, 12 children were identified during a 3-year period. The primary care population base was approximately 10,000 children with an estimated 3,200 children between 3 and 12 years (personal communication Michael Pichichero, MD). The incidence of PANDAS can be estimated to be 4 per 3,200 children/year or approximately 1 per thousand from this study.

The overall incidence of childhood onset OCD in the population of children less than 10 years of age varies from approximately 2%–4% (6). Swedo has speculated that as many as 25% of these cases may be related to GAS, for an incidence of 5–10 per 1,000 children (7). In contrast, a study by Jaspers-Fayer et al, reported that 5% of their population of children with OCD fit criteria for PANDAS or PANS (8). On a common website for parents, it is estimated that PANDAS affects 1 in 200 children (9).

Although a prospective study examining the incidence of PANDAS and PANS would be most desirable, a prospective strategy may not provide representative results during the current COVID pandemic and the immediate post-COVID period; masking and distancing may alter the natural history of those infections and other triggers that precede the onset of these syndromes. Accordingly, we embarked on a retrospective review for the calendar years 2017–2019 at three different academic medical centers. Our goal was to determine the incidence of PANDAS and PANS in several geographically and socioeconomically diverse primary care populations of children.

## 2. Methods

A retrospective investigation was undertaken within the primary care populations at the University of Wisconsin Hospital and Clinics (Madison, Wisconsin and the surround), Arkansas Children's Hospital (Little Rock, Arkansas and the surrounding geographic area) and the Children's Hospital at Dartmouth (Lebanon, New Hampshire and the surrounding geographic area). To identify cohorts of children who received primary care at each health care system for each of the study years, a proxy definition was used based on children who had completed at least one well-child visit any time during the study period. For the University of Wisconsin and Dartmouth locations, the study period was 1/1/2017–12/31/2019, while for the Arkansas location, the study period was defined as 1/1/2018–12/31/2019 due to limited data access for the calendar year 1/1/2017–12/31/2017. Institutional review board approval was obtained for each institution.

### 2.1. Target population

Each institution extracted data from their respective electronic health record of all children aged 3–12 years who had received at least one well-child visit at the institution at any time during the study period. A well-child visit was defined as either an encounter for routine child health examination with abnormal findings (Z00.121) or an encounter for routine child health examination without abnormal findings (Z00.129).

For each patient in the primary care population, the data collected included date of birth, sex, first (listed) race, ethnicity, zip code, and state. Well-child visit information included visit date and visit primary payer financial class.

### 2.2. Potential cases

To facilitate the review of patient charts, potential cases of PANS and PANDAS were identified from the overall primary care population. A case could be identified in primary care clinic, specialty clinic, emergency department or urgent care. **Potential cases** were defined as children who were aged 3–12 years old at the time they received one of five new diagnoses during the study period: avoidant/restrictive food intake disorder (ICD-10-CM: F50.82), other specified eating disorder (ICD-10-F50.89), separation anxiety disorder of childhood (ICD-10-CM:F93.0), obsessive-compulsive disorder (ICD-10-CM:F42.*), or other specified disorders involving the immune mechanism, not elsewhere classified (ICD-10-CM: D89.89). Potential cases could not have any of the five diagnoses from January 2007 to the start of the organization's study period, as the intent was to identify first presentation. The diagnosis of tic disorder was not used to identify potential cases because it is very common and usually self-limited. Separation anxiety of childhood was included to cast a slightly broader net as it often accompanies OCD and is a common supporting criterion in patients with PANS.

For each potential case, the first and last dates of any of the five diagnoses were collected. The diagnosis could have been entered by the primary care physician, neurologist, psychiatrist or other health care provider. The medical record of each potential case was reviewed from the time of the first presentation which was recorded as the age at presentation. The abstracted information included demographics (age, sex, location), date of onset of symptoms and whether criteria for PANS or PANDAS were fulfilled as defined in the next paragraph.

### 2.3. Actual cases

All **actual** cases were reviewed by two investigators. An actual case was identified as being consistent with **PANDAS** if the following criteria were met: (1) presence of OCD and/or tic disorder, (2) prepubertal onset, (3) acute and severe onset and dramatic exacerbations of symptoms, (4) presence of neurologic abnormalities during symptom exacerbations and (5) a temporal relationship between GAS infections and exacerbations of symptoms (positive culture, rapid test or serology) (1). Tics were included in the actual cases found in our study but were not included as a diagnostic code to identify cases in the incidence data and analysis. An **actual** case was identified as being consistent with **PANS** if the following criteria were met: abrupt, dramatic onset (within 48 h) of (1) OCD or (2) severely restricted food intake and (3) concurrent abrupt onset of additional severe neuropsychiatric symptoms from at least 2 of the following 7 categories: (1) anxiety; (2) emotional lability and/or depression; (3) irritability, aggression, and/or severe oppositional behaviors; (4) developmental regression; (5) deterioration in school performance; (6) sensory or motor abnormalities, including heightened sensitivity to sensory stimuli, hallucinations, dysgraphia, complex motor, and/or vocal tics; (7) somatic signs and symptoms, including sleep disturbances, enuresis or urinary frequency and the requirement that symptoms are not better explained by a known neurologic or medical disorder (4).

### 2.4. Statistical analysis

Demographic characteristics of the study population (overall and stratified by study site) and cases were summarized in terms of frequencies and percentages or means and standard deviations. Comparisons of demographic distributions between study sites were conducted using chi-square analysis. Frequencies and percentages of diagnostic symptoms of the cases were reported. Overall estimated incidence rates of PANDAS/PANS cases (per 100,000) were reported and stratified by demographic characteristics, along with the corresponding two-sided 95% confidence intervals (CIs) which were constructed using the Clopper-Pearson method. Comparisons of incidence rates between study sites, gender and study year were performed by calculating ratios of predicted probabilities derived from fitting corresponding logistic regression models. Due to the small number of actual cases, Fisher's exact test was used to compare incidence rates of actual cases between study sites, gender and study year. Pooled estimates of incidence rates of potential and actual cases and corresponding 95% CIs across the three study sites and across the three study years were obtained using a fixed effects model or random effects model with the inverse variance weighting method (10). Heterogeneity of incidence rates among sites was quantified by calculating the *I*^2^ statistics. All reported *p*-values are two-sided and *P* < 0.05 was used to define statistical significance. Statistical analyses were conducted using SAS software (SAS Institute, Cary NC), version 9.4. Calculation of pooled rates were conducted using R software (Foundation for Statistical Computing, Vienna, Austria, version 3.5.1) meta package.

## 3. Results

The combined primary care study population consisted of 95,498 unique subjects across the three study sites. The majority of the study population was non-Hispanic Caucasian (85%), 48% was female and the mean age was 7.1 (SD 3.1) years (Table 1). There was a total of 357 potential cases and 13 actual cases across the three geographic areas. The mean age of the potential cases was 7.5 (SD 2.4) years, 93% were non-Hispanic Caucasian and 49% were female. Significant differences in the distribution of gender, race, ethnicity and insurance type were detected among the three study sites (Table 2). Arkansas Children's Hospital serves a much larger population of underrepresented minority children (Black and Hispanic) who are publicly insured than either the Children's Hospital at Dartmouth or the University of Wisconsin. There was substantial heterogeneity in the symptom distributions of potential cases between study sites (Table 3). Across all three sites, the most common symptom was separation anxiety disorder of childhood (57%), 22% OCD, other specific eating disorder (15%), other specific disorders involving the immune system (7%) and avoidant/restrictive food intake disorders (3%). The most common symptom of the actual cases was OCD which was identified as the major symptom in 11 out of 13 subjects (85%). The mean age of the 13 actual cases was 6.0 (SD 1.8) years, 46% were female; 100% were non-Hispanic Caucasians, 85% had a commercial health insurance and 6 of 13 (46%) had a definite relationship to GAS. Details of the 13 actual cases are shown in Table 4.

**Table 1:** Demographics of primary care population and cases.

	Primary care population *N* = 95,498		Potential cases *N* = 357		Actual cases *N *= 13	
	*N*	%	*N*	%	*N*	%
Study site						
Arkansas Children's Hospital	24,434	26	49	14	0	0
Children's Hospital at Dartmouth	35,021	37	164	46	9	69
University of Wisconsin Hospital & Clinics	36,043	38	144	40	4	31
Gender						
Female	45,830	48	174	49	6	46
Male	49,663	52	183	51	7	54
Race						
Asian	4,069	4	6	2	0	0
Black/African American	15,270	16	32	9	0	0
White	65,157	68	303	85	13	100
Other	8,397	9	11	3	0	0
Unknown	2,605	3	5	1	0	0
Ethnicity						
Non-Hispanic	80,696	85	331	93	13	100
Hispanic	12,866	13	21	6	0	0
Unknown	1,936	2	5	1	0	0
Insurance type						
Commercial	55,591	42	226	63	11	85
Public	39,907	58	131	27	2	15
Study Year						
2017	41,102	27	149	42	4	31
2018	56,230	37	109	31	4	31
2019	56,562	37	99	28	5	38
	Mean	SD	Mean	SD	Mean	SD
Age (years)	7.1	3.1	7.5	2.4	6.0	1.8

**Table 2:** Demographic characteristics of primary care population, stratified by study site.

	Arkansas Children's Hospital *N* = 24,434		Children's Hospital at Dartmouth *N* = 35,021		University of Wisconsin Hospital & Clinics *N* = 36,043		
	*N*	%	N	%	*N*	%	*p*-value
Gender							
Female	11,552	47	16,919	48	17,359	48	0.0329
Male	12,881	53	18,102	52	18,680	52	
Race							
Asian	315	1	1,156	3	2,598	7	<0.0001
Black/African American	10,961	45	1,051	3	3,258	9	
White	5,085	21	30,804	88	29,268	81	
Other	7,932	32	157	0	308	1	
Unknown	141	1	1,853	5	611	2	
Ethnicity							
Non-Hispanic	16,202	66	31,675	90	32,819	91	<0.0001
Hispanic	7,506	31	2,366	7	2,994	8	
Unknown	726	3	980	3	230	1	
Insurance type							
Commercial	4,338	18	21,961	63	29,292	81	<0.0001
Public	20,096	82	13,060	37	6,751	19	

**Table 3:** Symptoms of potential cases.

	ACH		Dartmouth		UWHC		Combined	
	*N*^b^ = 49		*N*^b^ = 164		*N*^b^ = 144		*N*^b^ = 357	
	*N* ^c^	%^d^	*N* ^c^	%^d^	*N* ^c^	%^d^	*N* ^c^	%^d^
Avoidant/restrictive food intake disorder	1	2	4	2	4	3	9	3
Other specific eating disorder	24	49	30	18	0	0	54	15
Separation anxiety disorder	20	41	53	32	130	90	203	57
Obsessive-compulsive disorder	0	0	69	42	8	6	77	22
Other specific disorders^a^	4	8	9	5	12	8	25	7

^a^ Involving the immune mechanism, not elsewhere classified.

^b^ Number of unique subjects.

^c^ Frequency of symptom.

^d^ Percentage of unique subjects with symptom.

**Table 4:** Characteristics of cases of PANDAS/PANS at 3 academic centers during 2017–2019.

Age at presentation (years)^c^	Race C = Caucasian	Gender	Major symptom	Previously normal or previous mental health problem	Group A strep yes/no	Insurance (public/ commercial)	Year at presentation
3–7	C	Male	Food Avoidance	History of ADHD^b^ and autism	No	Commercial	2017
5–9	C	Male	OCD^a^	Previously normal	Yes	Commercial	2017
5–9	C	Male	OCD/Tics	Previously normal	Yes	Public	2018
3–7	C	Female	OCD/Food avoidance	Previously normal	No	Commercial	2019
6–10	C	Female	OCD	History of anxiety	No	Commercial	2019
3–7	C	Female	Food Avoidance	Previously normal	No	Commercial	2018
3–7	C	Male	OCD	Previously normal	No	Commercial	2019
4–8	C	Female	OCD	Previously normal	No	Commercial	2018
5-9	C	Female	OCD	Previously normal	No	Public	2019
6–10	C	Male	OCD	Previously normal	Yes	Commercial	2017
4–8	C	Female	OCD	Previously normal	Yes	Commercial	2017
3–7	C	Male	OCD	Previously normal	Yes	Commercial	2018
5–9	C	Male	OCD	Previously normal	Yes	Commercial	2019

^a^ OCD, obsessive-compulsive disorder.

^b^ ADHD, attention deficit hyperactivity disorder.

^c^ Ranges used to protect confidentiality.

The incidence rates of potential cases across the three geographic areas in the US ranged from 201 (95% CI: 152–265) to 468 (95% CI: 402–545) per 100,000 (Table 5). The heterogeneity of incidence rates of potential cases between sites was large with an *I*^2^ of 93%. The pooled estimate of the cumulative incidence rate of potential cases across the three geographic areas over the 3-year surveillance period was 340 (95% CI: 206–547) per 100,000. Incidence rates of potential cases were similar between females vs. males (RR 1.0, 95% CI: 0.8–1.3) and health insurance type (commercial vs. public, RR = 1.2, 95% CI: 1.0–1.5). The annual incidence rate of potential cases was significantly higher in 2017 when compared to 2019 (RR 2.1, 95% CI: 1.6–2.7, *p* < 0.0001). The pooled estimate of the cumulative incidence rate of actual cases across the three sites over the 3-year surveillance period was 14 (95% CI: 6–37) per 100,000 with an *I*^2^ of 55%, indicating a substantial amount of heterogeneity between geographic areas (Table 5). There were no statistically significant differences in the incidence rates of actual cases observed between males vs. females, insurance type or study year. However, all actual cases were

**Table 5:** Incidence rates of potential cases and actual cases, stratified by demographic characteristics and pooled estimates across the three study sites (geographic areas).

	Potential cases			Actual cases		
	Incidence rate^a^ per 100,000 (95% CI)	Rate ratio (95% CI)	*p*-value	Incidence rate^a^ per 100,000 (95% CI)	Rate ratio (95% CI)	*p*-value^b^
Study site						
ACH^f^	201 (152–265)	0.5 (0.4–0.7)	<0.001	0 (0–16)	0.0	0.1528
Dartmouth	468 (402–545)	1.2 (0.9–1.5)	0.1643	26 (14–49)	2.3 (0.7–7.5)	0.1738
UWHC^g^	400 (339–470)	Ref^h^		11 (4–29)	Ref	
Pooled estimate^c^	340 (206–547)	*I*^2 ^= 93%^e^		14 (6–37)	*I*^2 ^= 55%^e^	
Study year						
2017	363 (309–425)	2.1 (1.6–2.7)	<0.001	10 (4–25)	1.1 (0.3–4.1)	0.9999
2018	194 (161–234)	1.1 (0.8–1.5)	0.4620	7 (3–18)	0.8 (0.2–3.0)	0.9999
2019	175 (144–213)	Ref		9 (4–21)	Ref	
Pooled estimate^d^	232 (148–363)	*I*^2 ^= 95%^e^		9 (5–15)	*I*^2 ^= 0%^e^	
Gender						
Female	380 (327–440)	1.0 (0.8–1.3)	0.7776	13 (6–29)	0.9 (0.3–2.7)	0.7827
Male	368 (319–426)	Ref		14 (7–29)	Ref	
Insurance type						
Commercial	407 (357–463)	1.2 (1.0–1.5)	0.0515	20 (11–35)	3.9 (0.9–17.8)	0.0876
Public	328 (277–389)	Ref		5 (1–18)	Ref	

^a^ Cumulative incidence rates for study site, insurance type and gender over the three years surveillance period, annual incidence rates for study year.

^b^ Fisher's exact test.

^c^ Pooled estimate of cumulative incidence rate over the surveillance period across the three sites using a random effects model.

^d^ Pooled estimate of annual incidence rates across the three years using a random effects model for potential cases and fixed effects model for actual cases.

^e^ *I*^2^ of 0%–30% indicates low level of heterogeneity, 30%–50% indicates moderate level of heterogeneity, 50%–75% indicates substantial heterogeneity, >75% large level of heterogeneity.

^f^ Arkansas Children's Hospital.

^g^ University of Wisconsin Hospital and Clinics.

^h^ Ref = reference population which is University of Wisconsin.

Caucasians, resulting in incidence rate estimates of 16 (95% CI: 9–28) for Caucasians vs. 0 (95% CI: 0–30) per 100,000 for non-Caucasians. The pooled estimate of the annual incidence rate across the three study years was 8.5 (95% CI: 5–15) per 100,000 or 1 in 11,765 children.

## 4. Discussion

This retrospective study performed in three geographically and demographically diverse sections of the US showed that the estimated annual incidence of PANDAS/PANS was 1 in 11,765 children between 3 and 12 years of age with considerable place-to-place variation. Tic disorders was not used as a diagnostic code to identify cases of PANDAS. The mean age was 6.0 years; 46% of cases were related to GAS and the most common presenting symptom complex was with OCD.

The basic hypothesis underlying the concept of PANDAS by Swedo is that it represents a nonsuppurative complication of infection with GAS (on the basis of molecular mimicry) similar to, but different than, acute rheumatic fever (1). However, it is the very existence of Sydenham chorea, accompanied by severe OCD in some cases, that provides the biologic plausibility for the entity of PANDAS, i.e., why not OCD without the movement disorder of chorea as a manifestation of a presumed autoimmune disorder (11, 12). This hypothesis, which includes molecular mimicry and anti-neuronal autoantibodies, is supported by the fact that Sydenham chorea and PANDAS are strikingly similar with overlapping symptoms. The two diseases can be identified by testing for a group of cross-reactive anti-neuronal autoantibodies which are elevated when symptoms are present and decrease during improvement (13).

The controversy surrounding PANDAS/PANS has not served the primary care provider very well, leaving them confused as to its importance and existence as a diagnostic entity. Studies intended to clarify the role of GAS as an antecedent event in cases of PANDAS have resulted in differing results, with some supporting its role (14–16) and others not (17–19). Although the relationship of GAS pharyngitis as an antecedent event to acute rheumatic fever is well-established it is qualified by at least two factors. Not all GAS cause acute rheumatic fever and not all individuals have the genetic predisposition to develop acute rheumatic fever even when infected with a rheumatogenic strain of streptococcus (20, 21). These factors may explain, at least in part, the discrepant results observed by investigators when attempting to discern the relationship between GAS and PANDAS/PANS. The definitive diagnosis of PANS and/or PANDAS requires a comprehensive gathering of historical information supplemented by a variable laboratory evaluation for potential infectious triggers at the time of presentation. If acuity of onset of neuropsychiatric symptoms and potential infectious triggers are not explored the diagnosis may be missed. This information will guide treatment that may differ if symptoms are not post-infectious.

Although we elected not to study the incidence of PANDAS\PANS during COVID, there have been multiple reports of SARS-COV-2 related cases of PANS as well as other post-infectious COVID-19 related neurologic syndromes (22–25). This is not surprising as there are many infectious disease agents that have been associated with the development of PANS (26).

In our study, 7 of 13 (54%) children with PANS/PANDAS were male which was not significantly different than the incidence in girls. However, the numbers of actual cases are small, and 5 of 6 children with PANDAS were boys, similar to the report by Swedo et al. (1). It is of interest that when Cox et al., were exploring the prevalence of antineuronal antibodies in a large group of children with a history of streptococcal infection and tic and/or OCD they found the highest association with a history of GAS in children with both disorders compared to either tics or OCD alone (27). The demonstration of antineuronal antibodies was similar to their previous demonstration of antibodies in both Sydenham chorea and PANDAS (28, 29).

There are no previous examinations of the estimated incidence of PANDAS/PANS in the US or any other population. Popular estimates relate to the epidemiology of OCD and what proportion may be attributable to GAS as an instigating event (8). Other estimates are not based on obvious sources of information (9). GARD (Genetic and rare diseases information center) is a program of the National institutes of Health that provides free access to reliable and easy to understand information about genetic and rare diseases. They list PANDAS and state that the population estimate section is “in development” (30). For comparison, the estimated incidence of autoimmune encephalitis in Olmstead County, Minnesota in 2014 was shown to be 137/100,000 or 1 in 7,299 (31).

We attempted to identify potential cases of PANDAS and PANS by searching for new diagnoses of OCD, food avoidance/restriction, other specified eating disorder, separation anxiety of childhood and other specified disorders of the immune system not otherwise classified in our primary care population. The proportion of the diagnoses shown at each institution were surprisingly different (Table 3). Arkansas had no cases of obsessive-compulsive disorder perhaps contributing, at least in part, to these differences, which otherwise remain unexplained but portend the substantial differences in estimated incidence at the three centers. Often potential cases identified on the basis of a new diagnosis of separation anxiety were missing key criteria for the diagnosis of PANDAS/PANS. Many patients with food restriction/avoidance or other specified eating disorders were categorized as anorexia nervosa or pica. Ten patients with OCD were lacking a description of the acuity of onset of symptoms and could not be classified as PANS or PANDAS. The complete absence of cases at Arkansas was unexpected but perhaps reflects either the absence of “pandogenic” streptococci during the study period, or potential differences in risk of disease related to underrepresented minority status. The demographics of the population studied at Arkansas was also different from the others with respect to insurance and race.

Although our results indicate that PANDAS/PANS is a rare disorder with substantial heterogeneity across geography and time, we must consider potential causes of underdiagnosis or how our methods may have missed cases. First, there are physicians who are unaware of this diagnosis and therefore do not associate any of the symptoms which commonly signal the disorder such as anxiety, food restriction or other specified eating disorders or OCD as possibly triggered by infectious agents. On some occasions, the physician may simply not inquire about a particular symptom. Since our search for cases is dependent on finding these codes for symptoms, their absence from the chart does not assure that the question was asked. When physicians do identify a symptom, they may not elicit a thorough history which indicates that the onset of the symptom was uncommonly abrupt. They will be unlikely to search for triggers in a child who presents with debilitating urinary frequency and a negative urinalysis; this highlights the impossibility of retrospectively determining whether symptom onset was preceded by an asymptomatic GAS infection. Physicians may fail to recognize other behaviors as consistent with OCD, thereby again raising the important question of the ability to study the incidence of these conditions retrospectively. In addition, there are some practitioners who view the published evidence supporting PANDAS/PANS as unconvincing and who therefore may be less likely to pursue any supporting evidence such as cultures or serology in children with the acute onset of neuropsychiatric symptoms.

We decided not to use the diagnostic code for tics to identify cases of PANDAS despite the fact that tics are a major criterion upon which this diagnosis is based. The decision was predicated on the very common occurrence of tics, almost all of which have their onset in childhood, which, it is safe to say, is upward of a least 10%–20% of children depending on geography, age and study methods (32). We were concerned that including tics would introduce excessive data which was likely to be incomplete and increase the noise/signal ratio in an unhelpful way. Some patients detected through diagnostic code D89.89 acquired this code, which is used for patients with PANDAS, by their presentation with tics and otherwise fulfilled diagnostic criteria. We recognize that not using the code for tics may have resulted in an underestimate of the cases of PANDAS and be a limitation of this study. Several other limitations should be noted. Our proxy definition for determining the cohort of children receiving primary care at each institution in each study year may have overestimated or underestimated the number of person-years in our denominator(s) which in turn may have resulted in biased year-specific estimates of incident rates. Given the large data base, we doubt this resulted in a significant difference. In addition, the age group studied, 3–12 years of age, was selected to avoid missing younger children with PANDAS. However, there is a slight skewness in the age distribution with over-representation of the younger age groups which may bias results in favor of a lower estimate. In addition, not extending the upper bound of age to 18 years may miss patients with PANS.

What we have learned from this pilot study is that PANS and PANDAS appear to be uncommon diagnoses in children in these areas of Wisconsin, Arkansas and New Hampshire who seek care at academic medical centers. In addition, we learned from careful review of our potential cases that some patients who ultimately came to hold a diagnosis of PANS/PANDAS did not qualify for inclusion as a “case” in this study because of an initial lack of evaluation or incomplete evaluation. While we await additional prospective studies to help determine the precise incidence of these diagnoses and their natural history, although they are likely rare, the clinician should be prepared to contemplate and pursue a PANS/PANDAS diagnosis when clinical symptoms are suggestive of a post-infectious potentially autoimmune neuropsychiatric syndrome.

## Data Availability

The data analyzed in this study is subject to the following licenses/restrictions: existing medical records from three different academic medical centers were abstracted, reviewed and analyzed. Requests to access these datasets should be directed to Jens Eickhoff Eickhoff@biostat.wisc.edu.
